# Influence of Maternal Adipokines on Anthropometry, Adiposity, and Neurodevelopmental Outcomes of the Offspring

**DOI:** 10.3390/ijms252111655

**Published:** 2024-10-30

**Authors:** Jorge Valencia-Ortega, Andrea Castillo-Santos, Miranda Molerés-Orduña, Juan Mario Solis-Paredes, Renata Saucedo, Guadalupe Estrada-Gutierrez, Ignacio Camacho-Arroyo

**Affiliations:** 1 Unidad de Investigación en Reproducción Humana, Instituto Nacional de Perinatología-Facultad de Química, Universidad Nacional Autónoma de México, Mexico City 11000, Mexico; j.valencia.o@hotmail.com; 2 Department of Reproductive and Perinatal Health Research, Instituto Nacional de Perinatología Isidro Espinosa de los Reyes, Mexico City 11000, Mexico; andreaocassan@gmail.com (A.C.-S.); mirandamoleres@gmail.com (M.M.-O.); juan.solis@inper.gob.mx (J.M.S.-P.); 3 Unidad de Investigación Médica en Enfermedades Endocrinas, Hospital de Especialidades, Centro Médico Nacional Siglo XXI, Instituto Mexicano del Seguro Social, Mexico City 06720, Mexico; renata.saucedo@imss.gob.mx; 4 Department of Immunobiochemistry, Instituto Nacional de Perinatología Isidro Espinosa de los Reyes, Mexico City 11000, Mexico; guadalupe.estrada@inper.gob.mx

**Keywords:** adipose tissue, fetal programming, neonatal outcomes, adiponectin, leptin

## Abstract

Pregnancy is distinguished by a multitude of intricate interactions between the mother and the new individual, commencing at implantation and persisting until the maturation and integration of the fetal apparatus and systems. The physiological increase in fat mass during pregnancy and the association of maternal obesity with adverse neonatal outcomes have directed attention to the study of maternal adipokines as participants in fetal development. Interestingly, maternal concentrations of certain adipokines such as adiponectin, leptin, tumor necrosis factor-alpha, and interleukin-6 have been found to be associated with offspring anthropometry and adiposity at birth and at three months of age, even with neurodevelopmental alterations later in life. This is partly explained by the functions of these adipokines in the regulation of maternal metabolism and placental nutrient transport. This review compiles, organizes, and analyzes the most relevant studies on the association between maternal adipokines with anthropometry, adiposity, and neurodevelopmental outcomes of the offspring. Furthermore, it proposes the underlying mechanisms involved in this association.

## 1. Introduction

Pregnancy is characterized by diverse and complex interactions between the mother and the new individual that begin at implantation and continue through embryonic development, organogenesis, and the maturation and integration of fetal apparatus and systems. Each of these stages exhibits an accentuated cellular proliferation and programming, which in turn represents a period of vulnerability for the fetus [1].

The developmental origins of health and disease (DOHaD) hypothesis states that the early life conditions of an individual (periconceptional, fetal, and early childhood stages) determine health or disease later in life [2]. The exposition to adverse factors during pregnancy compromises proper fetal development, leading to metabolic or structural alterations [1]. Among the most common negative factors in pregnancy associated with adverse neonatal and infant outcomes is maternal obesity, which is characterized by an increase in fat mass [3]. These findings have given rise to the idea that adipokines produced by the adipose tissue influence fetal development due to their ability to modulate maternal and placental metabolism. The placenta is an adipokine-producing organ, and excessive maternal weight deregulates its function [4].

Several studies have demonstrated an association between maternal obesity, adipokine dysregulation, and adverse neonatal outcomes. However, it is noteworthy that maternal adipokine concentrations have also been linked to certain neonatal characteristics such as weight and length at birth, even without excessive maternal weight or pregnancy complications [5,6,7,8,9,10,11,12]. This indicates that maternal adipokines may exert a physiological influence on fetal programming.

Based on this, this review aims to organize and analyze the most relevant studies on the association between maternal adipokines with anthropometry, adiposity, and neurodevelopmental outcomes of the offspring.

## 2. Search Process

The following search terms were used by the authors to retrieve articles from Pubmed: “maternal metabolism”, “metabolism in pregnancy”, “placental metabolism”, “placental nutrient transport”, “adipokines”, “adipose tissue”, “maternal obesity”, “newborn outcomes”, “newborn anthropometry”, “birth weight”, “birth length”, “infant neurodevelopment”, “infant neurodevelopmental disorders”, and “newborn adiposity”. Only relevant English-language articles were chosen.

## 3. Maternal Metabolism Adaptation in Pregnancy and Placental Nutrient Transport

Pregnancy represents an energetic challenge for the maternal system, as it must sustain its basal metabolic functions and simultaneously supply nutrients to the fetus. This metabolic adaptation takes place in two distinct phases. The initial one, which occurs during the first half of pregnancy, is designated as the anabolic phase. This is typified by augmented energy storage, predominantly in the form of lipids, to fulfill the requirements of advanced pregnancy and lactation. The second phase, designated the catabolic phase, is distinguished by a notable decline in insulin sensitivity and an increase in maternal circulating concentrations of glucose and free fatty acids, which are mobilized to meet the energy demands of fetal growth, particularly pronounced in the second half of pregnancy [13].

Glucose serves as the primary energy substrate required by the feto-placental unit. This is perfectly coupled with progressive maternal insulin resistance, which is more pronounced in the third trimester of pregnancy when fetal growth accelerates markedly [14]. In this trimester, maternal fasting glucose concentrations are significantly reduced, insulin concentrations are more than tripled, and hepatic gluconeogenesis is increased. This increase in endogenous glucose production is an essential metabolic adaptation to this energetic challenge [15,16].

Concerning lipid metabolism, during the anabolic phase of pregnancy, high concentrations of estradiol, progesterone, and insulin promote the synthesis of fatty acids and the expression of lipoprotein lipase, facilitating the uptake of circulating triglycerides. As pregnancy progresses, concentrations of fatty acids, triglycerides, cholesterol, and phospholipids continuously increase [17]. In the catabolic phase, particularly during the third trimester of pregnancy, energy storage in adipose tissue decreases, while lipoprotein lipase activity, adipose tissue lipolysis, and free fatty acid concentrations increase. As a result, lipids become the mother’s primary energy source, whereas glucose and amino acids are mainly destined for the fetus [18].

The placenta has multiple functions, including protection from the maternal microenvironment and infection, establishment of immune tolerance, exchange of nutrients, gases, and wastes, and regulation of maternal metabolism [16]. These functions depend on several factors, including placental size and morphology, uteroplacental perfusion, growth factors, hormones, and other molecular processes involved in cellular transport [18]. In the placenta, maternal and fetal circulatory systems are separated by the placental barrier, formed by the syncytiotrophoblast and the fetal capillary endothelium. The syncytiotrophoblast layer has two membranes: the microvilli membrane (MVM), which is directed toward the intervillous space, and the basal membrane, which is immediately adjacent to the fetal capillaries. These membranes are the first players in the placental transport of nutrients [16].

Glucose transport occurs by facilitated diffusion through specific glucose transporter proteins (GLUTs) expressed in both syncytiotrophoblast membranes [19]. Given that maternal circulating glucose concentrations are higher than in the fetal circulation, net transport is to the fetus. There are 14 members of the GLUT family, and although several of them are variably expressed in the placenta, GLUT1 is considered its major transporter [20].

Amino acid transport occurs through active transport processes, accumulative transporters, or exchangers that are expressed in both syncytiotrophoblast membranes. Amino acid concentrations are higher in fetal plasma than in maternal plasma, suggesting active transport predominates [21]. The placenta expresses more than 15 amino acid transport systems. Some amino acids are transported by a single system, while others may be transported by multiple systems. The best-known systems are the A- and L-systems [22]. System A consists of a set of transporter proteins that facilitate the capture of small non-essential neutral amino acids such as alanine, glycine, and serine against their concentration gradient by cotransport with sodium into the cell. This system includes the solute carriers (SLCs) SLC38A1, SLC38A2, and SLC38A4, expressed in the MVM of term placentas. Interestingly, the activity of these transporters varies throughout pregnancy, with SLCA4 activity being more prominent in the first trimester, whereas SLCA1 activity increases at term [23]. The amino acids provided by system A are utilized by system L to be exchanged for long essential amino acids, thus allowing their transport against their concentration gradient. The L-system is a heterodimer formed by a light chain L-type transporter (such as SLCA5 and SLCA8) covalently attached to a heavy chain (SLCA2), and exchanges aromatic and branched essential amino acids in a sodium-independent manner [24]. In term placentas, SLCA5 and SLCA8 are expressed in trophoblast; both are present in the MVM, and only SLCA8 is in the basal membrane [25].

It was thought that lipid transport, specifically free fatty acids, freely occurs in the placenta. However, it is now understood that, through the activity of lipoprotein and endothelial lipase enzymes, the placenta converts maternal lipids into free fatty acids, which are then captured and processed by trophoblast cells to meet their energetic demands to produce hormones and to transfer them to the developing fetus. Adequate lipid uptake from the early stages of pregnancy is essential to meet the metabolic demands of the developing placenta and embryonic organogenesis. In late pregnancy, there is an even greater demand for lipids to sustain fetal neurodevelopment and growth [26]. Consequently, as pregnancy progresses, metabolic adaptations in the mother and placenta favor lipid transport and accumulation of long-chain essential polyunsaturated fatty acids in the third trimester [27]. These fatty acids cannot be synthesized de novo; thus, the fetus exclusively obtains them from the mother. They are vital because being situated in the cell membrane, they serve as mediators of numerous processes, including metabolism, inflammation, placental aggregation, signal transduction, neurotransmission, and neurogenesis [28].

It is important to mention that the nutrient transport profile changes throughout pregnancy; for example, the expression of systems A and L varies as pregnancy progresses [21]. Evidence suggests that the placenta monitors and adapts to nutrient availability to support normal fetal growth [29]. This indicates that placental nutrient transport is a process that interacts with maternal metabolism and can change its dynamics in favor of fetal development.

## 4. Cellular and Molecular Mechanisms of Adipokines in Fetal Development

Some adipokine functions, which are detailed below, make it plausible to hypothesize that they participate in fetal development and programming. Only the functions of leptin, adiponectin, tumor necrosis factor-alpha, and interleukin-6 (IL-6) are described; because, as detailed later in the text, their maternal concentrations have been associated with neonatal characteristics. It is important to note that it is not possible to establish a direct connection between maternal concentrations of these adipokines with the anthropometry, adiposity, and neurodevelopment of the offspring. At present, the evidence only allows us to hypothesize that these adipokines influence the characteristics of the neonate through the regulation of maternal metabolism and placental transport of nutrients, which ultimately determine the availability of nutrients for fetal development. In some cases, adipokines induce apoptosis in placental cells, which could affect the proper functioning of placental tissue.

### 4.1. Leptin

Leptin is a 16-kDa peptide that regulates metabolism and body weight by acting in different hypothalamic regions to reduce food intake and increase energy expenditure [30]. It also modulates the immune response and participates in reproductive processes such as implantation and embryonic development [31,32]. Leptin exerts its effects through binding to leptin receptors (LepRs), with the long isoform of a LepRb emerging as the principal mediator of leptin signaling [33,34]. LepRbs are expressed in various organs and tissues, including the hypothalamus, lung, kidney, adipose tissue, endothelial cells, blood cells, stomach, muscle, liver, pancreatic islets, osteoblast, endometrium, placenta, and umbilical cord [35]. The soluble leptin receptor (sOB-R) plays a pivotal role in regulating free leptin concentrations, thereby modulating the availability of leptin to bind to leptin receptors on cells and exert its biological effects. In contrast to the typical effect of leptin on satiety, human pregnancy is characterized by a central resistance to leptin in the second trimester. This induces an increase in food intake to prevent maternal nutrient depletion, thereby allowing an increased nutrient supply to the growing fetus. This leptin resistance may be attributed to a reduction in hypothalamic LepRb expression [36].

Leptin is produced by adipose tissue, but in pregnancy, the placenta is the main leptin-producing tissue that contributes to both maternal and fetal concentrations throughout pregnancy. It is estimated that 95–98.4% of placental leptin is released into the maternal circulation, while only 5% or less enters the fetal circulation [37,38]. Maternal leptin concentrations progressively increase as pregnancy progresses, reaching concentrations two to three times higher than in non-pregnant women. After delivery, leptin concentrations rapidly decrease to pregestational levels. This hyperleptinemia state is considered essential for fetal development since leptin is involved in maternal glucose metabolism and placental nutrient transport, processes that ultimately regulate the mobilization of nutrients to the fetus [39].

Regarding glucose metabolism, leptin regulates insulin action and hepatic gluconeogenesis, affecting whole-body insulin sensitivity. It may contribute to insulin resistance by phosphorylating serine residues of insulin receptor 1 substrate (IRS1), downregulating insulin signaling. Leptin also acutely inhibits insulin secretion by pancreatic β-cells [40]. Although there is no consensus on the effect of leptin on hepatic gluconeogenesis, it has been observed that it can activate signal transducer and activator of transcription 3 (STAT3) and AMP-activated protein kinase (AMPK). Activation of STAT3 and AMPK has been reported to suppress gluconeogenesis [41]. It can be concluded that maternal leptin levels exert a significant influence on the availability of glucose to the developing fetus.

Concerning placental transport of nutrients, leptin participates in placental amino acid transport by stimulating system A through the JAK-STAT signaling pathway [42]. Furthermore, leptin has been demonstrated to reduce triglyceride and cholesterol levels in human placentas at term. Additionally, in both placenta and trophoblast cultures, it has been observed increasing glycerol release in a dose-dependent manner indicating lipolysis. Moreover, it has been demonstrated that leptin does not stimulate fatty acid beta-oxidation in BeWo cells. Therefore, the evidence suggests that leptin plays a role in the degradation of triglycerides to free fatty acids, which are the primary lipids transferred to the developing fetus [43].

It is known that fetal circulating leptin is mainly derived from fetal adipose tissue and that fetal leptin concentrations positively correlated with birth weight and gestational age. Moreover, umbilical cord leptin concentration is a marker of neonatal fat mass [44]. Because leptin cannot freely cross the placental barrier, it is generally accepted that there is no correlation between maternal and fetal leptin concentrations [45]. Nevertheless, a positive correlation between maternal serum and cord blood leptin concentrations has been reported. Higher cord leptin concentrations were observed in cases of excessive pre-pregnancy weight and excessive gestational weight gain, suggesting the possibility of a connection between maternal status and fetal leptin levels [46]. Nonetheless, other studies have not observed these associations [45,47].

### 4.2. Adiponectin

Adiponectin is an adipokine produced by adipose tissue and secreted into the bloodstream in four forms: trimers, hexamers, high molecular weight multimers, and the globular form [48]. Adiponectin has three distinct receptors: (1) AdipoR1, a high-affinity receptor for globular adiponectin with low affinity for the full-length form, which is ubiquitously expressed, although more abundantly in skeletal muscle; (2) AdipoR2, which mainly recognizes the full-length form and is primarily expressed in the liver; and (3) T-cadherin, which acts as a receptor for the hexameric and multimeric forms [49,50]. High molecular weight adiponectin is the most abundant isoform in the circulation and is considered the most biologically active isoform, particularly for its effect on insulin sensitivity [51]. Maternal concentrations of this adipokine decrease as pregnancy progresses, reaching its lowest levels in the third trimester when insulin resistance is greatest. It has been observed that maternal adiponectin concentrations are affected by BMI and ethnicity [52,53].

The influence of adiponectin on metabolism is profound. It improves insulin sensitivity through the promotion of fatty acid oxidation and glucose uptake in skeletal muscle, by inhibiting gluconeogenesis, lipogenesis, and inflammation in the liver, and by suppressing inflammation, promoting lipid metabolism, and facilitating glucose homeostasis in adipose tissue [54,55]. In pancreatic β-cells, adiponectin has been demonstrated to promote insulin secretion, reduce apoptosis, and enhance cellular survival and viability [56].

Regarding placental nutrient transport, adiponectin inhibits the expression of the major glucose transporters (GLUT1 and GLUT12) and sodium-coupled neutral amino acid transporters SNAT1, SNAT2, and SNAT4, all members of system A transporters, which may restrict the availability of nutrients for the fetus. Furthermore, adiponectin has been demonstrated to induce apoptosis in placental cells by enhancing the expression of the pro-apoptotic B-cell lymphoma-2-associated X protein and the tumor protein P53 gene expression, and by inducing the caspase activity, affecting the entire placental function [57].

### 4.3. TNF-α

During pregnancy, the immune system plays a pivotal role in maintaining the normal course of gestation. It is now established that reproductive success hinges on a delicate equilibrium between Th1, Th2, and Th17 cytokines and regulatory T cells [58]. TNF-α is a multifunctional Th1 cytokine and one of the most important inflammatory cytokines. It is mainly produced by macrophages, but also in adipose tissue and placenta. Its receptors are classified as tumor necrosis factor receptor 1 (TNFR1) or tumor necrosis factor receptor 2 (TNFR2). TNFR1 is expressed in all human tissues and serves as the primary signaling receptor for TNF-α. In contrast, TNFR2 is predominantly expressed in immune cells and plays a limited role in biological responses [59]. In addition to its pro-inflammatory function, TNF-α has been demonstrated to induce fever, apoptosis, and cachexia, as well as to inhibit tumorigenesis and viral replication. In pregnancy, TNF-α influences hormone synthesis (e.g., human chorion gonadotropin), placental architecture, and embryonic development [60]. Maternal TNF-α concentrations increase as pregnancy progresses [58].

TNF-α plays a pivotal role in the development of insulin resistance by reducing the expression of the insulin-regulated glucose transporter type 4 (GLUT4) in adipocytes and skeletal muscle. Furthermore, it induces serine phosphorylation of insulin receptor substrate-1 (IRS1), which inhibits insulin receptor activity [61]. Consequently, TNF-α concentrations throughout pregnancy may contribute to the physiological insulin resistance that permits glucose availability to the developing fetus.

TNF-α is essential for reproduction at the maternal–placental interface as it is involved in implantation and normal placental development. In addition, TNF-α has been shown to induce apoptosis in cultured trophoblast cells [62]. In BeWo cells, TNF positively regulates GLUT1 in a concentration-dependent manner [63]. Furthermore, this factor regulates placental amino acid transport, increasing the gene and protein expression of SNAT2 and upregulated SNAT1 protein expression in cultured human primary trophoblast cells [64]. In primary placental endothelial cells, TNF-α positively regulates endothelial lipase expression, activating the NF-κB pathway [65].

### 4.4. IL-6

IL-6 is a member of the IL-6 family of cytokines, and it participates in organ development, acute-phase response, metabolic regulation, inflammation and immune responses, and neural differentiation. These effects are exerted upon binding to its receptor IL-6R and subsequently activating the JAK-STAT pathway. IL-6 and other members of its cytokine family may exert both pro- and anti-inflammatory functions [66,67]. IL-6 expression is detected in different cell populations from the uteroplacental tissues during the first trimester of pregnancy, like in the decidua and placenta. IL-6 expression at the mRNA and protein levels was also shown in isolated first-trimester decidual natural killer cells, CD8+ T cells, and macrophages, as in cytotrophoblast cells, extravillous trophoblast cells, and primary decidual stromal cells; these last three group cells are shown to increase throughout pregnancy, especially in the second and third trimester [68]. This suggests that maternal IL-6 circulating levels should be elevated throughout pregnancy; however, there are no significant differences between trimesters, and even a decrease in the circulating IL-6 levels during gestation [69,70].

IL-6 has been demonstrated to induce insulin resistance by impairing the phosphorylation of the insulin receptor and insulin receptor substrate-1. This occurs through the induction of the expression of the suppressor of cytokine signaling 3, which has been identified as a potential inhibitor of insulin signaling [71].

IL-6 could alter the transfer of nutrients, hormones, or other key molecules to the fetus [62]. It has been seen that IL-6 also upregulates fatty acid uptake in human trophoblast cells, and it stimulates placental system A amino acid transport. It has been reported that STAT3 activation constitutes a critical mechanistic link between IL-6 and increased amino acid transport [72].

**Figure 1:**
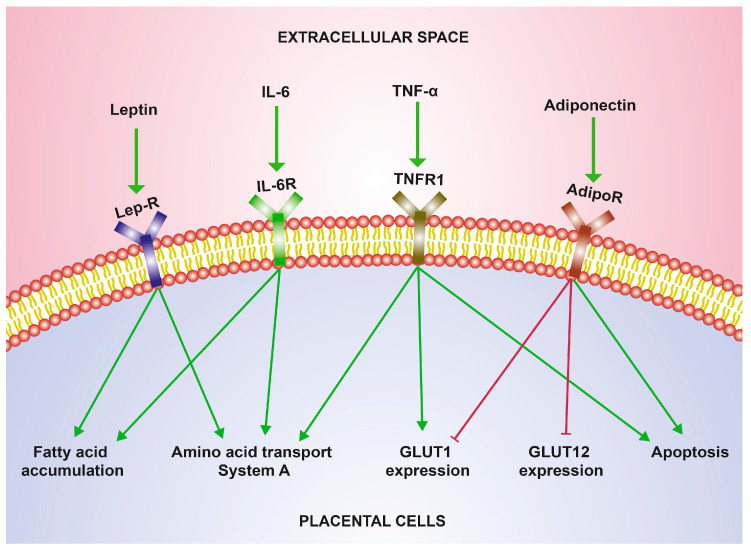
Effects of adipokines on placental nutrient transport. The main effects of leptin, IL-6, TNF-α, and adiponectin on placental mechanisms of glucose, amino acid, and lipid transport are shown (see the text for deeper details). Green arrowhead lines indicate stimulation and red T-shaped lines indicate inhibition. IL-6: interleukin-6; TNF-α; tumor necrosis factor-alpha; Lep-R: leptin receptor; IL-6R: IL-6 receptor; TNFR1: tumor necrosis factor receptor 1; AdipoR: adiponectin receptor; GLUT: glucose transporter.

In summary, leptin, TNF-α, and IL-6 may participate in progressive maternal insulin resistance in pregnancy, whereas the insulin sensitivity-enhancing effect of adiponectin diminishes as its maternal concentrations decrease. The effects of these adipokines on placental nutrient transport are summarized in Figure 1.

## 5. Associations Between Maternal Adipokine Levels and Anthropometry, Adiposity, and Neurodevelopmental Outcomes of the Offspring

The studies summarized in Table 1 show significant associations between maternal adipokine concentrations with different anthropometric outcomes of the neonate. Studies on maternal adipokines and neurodevelopmental outcomes are described later in the text.

The study by Sámano et al. [6] is notable for finding associations of maternal leptin concentrations with anthropometric characteristics of the infant. Positive correlations were observed between leptin maternal concentrations at 28 weeks of gestation and infant length at one, two, and three months of age. Moreover, at 32 weeks of gestation, leptin concentrations positively correlated with length at two months of age.

Importantly, in the study by Lekva et al. [9], it was observed that adiponectin concentrations decreased more in women giving birth to large for gestational age newborns than those with small or adequate for gestational age newborns, while leptin concentrations in early pregnancy (14–16 weeks) were higher in women with large for gestational age infants than in those with adequate for gestational age infants.

Anderson-Hall et al. [11] conducted multivariate regressions to evaluate the association between maternal variables and neonatal fat mass at 12 weeks of age. Their findings revealed that in women with normal weight, the maternal concentrations of soluble leptin receptor in trimesters 2 and 3 were negatively associated with neonatal fat mass; even the concentrations of this receptor throughout pregnancy were negatively correlated with the change in fat mass between weeks 1 and 12. In contrast, in women with pregestational obesity, maternal adiponectin concentrations were negatively associated with the change in fat mass.

In summary, the adipokines most studied in their relation to the anthropometric characteristics of the newborn are leptin and adiponectin. Although a significant relationship between these adipokines and birth weight is observed, there is no consensus on whether it is positive or negative. In the rest of the newborn characteristics, there are very few studies, so that it is not possible to highlight any trend. It is remarkable that, except for the study conducted by Perichart-Perera et al. [7], and Anderson-Hall et al. [11], all the findings included in Table 1 involved women with normal or excessive pregestational weight in their analyses of the associations between adipokines and anthropometric characteristics and neonatal adiposity.

The prevalence of mental, behavioral, and neurodevelopmental disorders in children has considerably increased in recent decades. In the United States, it is estimated that 15% of children between the ages of 2 and 8 have one or more neurodevelopmental disabilities [73]. A meta-analysis evaluated the impact of maternal obesity and gestational weight gain on the neurodevelopment of children aged 1 to 9 years and found that children born to women with pregestational overweight or obesity were at increased risk of neurodevelopmental disorders. Specifically, children of women with pregestational obesity were at increased risk of attention-deficit/hyperactivity disorders, autism spectrum disorders, developmental delay, and emotional/behavioral problems [74]. However, other studies have not found a relationship between maternal obesity and neurodevelopmental outcomes in children [75,76]. Because results are inconsistent, more research is needed to determine the extent to which maternal metabolic conditions may affect the child´s neurodevelopment.

It is noteworthy that one study has indicated that elevated maternal adiponectin concentrations in the second trimester of pregnancy are associated with a reduced risk of autism spectrum disorders in the offspring [77]. This observation is consistent with the established fact that the second trimester of pregnancy is characterized by enhanced neurogenesis and synaptogenesis in the fetal nervous system [78].

In murine models, it has been demonstrated that IL-6 signaling in the placenta is required for relaying inflammatory signals to the fetal brain, which are relevant to neurodevelopmental disorders [79,80].

Further research is required to elucidate the impact of maternal adipokines on neurodevelopmental outcomes in offspring. As previously discussed, placental lipid transport is essential for optimal fetal neurodevelopment. Consequently, investigating the influence of maternal adipokines on placental lipid transport and its association with neurodevelopmental modifications in the infant represents a promising avenue for future research.

## 6. Findings in Animal Models

Some studies in animals have indicated that maternal leptin concentrations may play a role in fetal programming. At gestational day 13, fetal weights of mice with the A^y^ mutation (which increases blood leptin concentrations during pregnancy) were observed to be lower than in the control group [81]. Furthermore, leptin-treated mice and rats displayed inhibition of fetal growth [82,83,84]. Conversely, chronic infusion of adiponectin in normal-weight pregnant mice has been demonstrated to result in a reduction in fetal weight [85]. This finding has been corroborated through the use of maternal knockout models for adiponectin, which have revealed the occurrence of fetal overgrowth [86,87]. It is notable that maternal adiponectin supplementation was able to reverse the deleterious effects of maternal obesity (which is characterized by maternal hypoadiponectinemia) on fetal overgrowth [88]. It has been reported that IL-6 administration to pregnant mice results in smaller newborns [89]. There are no studies that evaluated the effect of TNF-α administration in pregnant mice on newborn outcomes.

Interestingly, some studies in murine models show that maternal adipokine concentrations have an effect on the metabolic characteristics of the offspring in adulthood. Dumolt et al. [90], observed that male and female offspring of obese mice exhibited glucose intolerance and insulin resistance at 6 and 9 months of age, but these metabolic disturbances in offspring were prevented by normalization of maternal adiponectin levels in late pregnancy. Thus, maternal adiponectin is a critical link between maternal obesity and the development of metabolic disease in offspring. Denisova et al. [83], demonstrated that leptin administration in pregnant females has a sex-specific effect on the metabolism of the adult offspring: increasing resistance to obesity only in male offspring through a shift in food preference in favor of a balanced diet and maintenance of insulin sensitivity in muscle tissues. These findings raise the possibility that maternal adipokine concentrations may play a role in the development of metabolic alterations in adulthood in humans; however, no studies have evaluated this hypothesis.

## 7. Perspectives

Multicenter studies with better control of confounding variables, a longitudinal analysis of maternal adipokine concentrations, and a more robust statistical analysis, enabling the observation of the effects of pregestational maternal nutritional status, gestational weight gain, and even the consideration of biochemical markers that provide insight into metabolic status beyond body weight are needed. It is also imperative to study other adipokines that exert influence over maternal metabolism and/or placental nutrient transport. Furthermore, the potential association between maternal adipokine levels and neurodevelopmental disorders in infants has yet to be elucidated. While not the focus of this review, it is important to note that the placenta itself is a significant source of adipokines. Therefore, further investigation into the local regulation of adipokines represents a promising area for future research.

## 8. Conclusions

Adipokines are essential in fetal programming, modulating maternal metabolism and placental nutrient transport. Evidence indicates that maternal leptin and adiponectin levels are associated with neonatal anthropometry, while leptin and IL-6 levels are associated with neonatal adiposity and adiponectin levels are linked to alterations in the offspring’s neurodevelopment. However, some findings are inconsistent, and further research is required to corroborate these results.

## Figures and Tables

**Table 1:** Main characteristics and findings of studies on maternal adipokine concentrations and their relation to anthropometry and adiposity of the neonate.

Author (Year)	*n*	Adipokine	Gestational Age at Adipokine Measurement	Bivariate Correlation	Multivariate Association	Comments
Birth weight (g)						
Vernini et al. (2016) [5]	72 mother–newborn pairs	Adiponectin	37–38 weeks	+ r = 0.23	NS	Adiponectin levels were negatively correlated with gBMI
Sámano et al. (2017) [6]	168 dyads	Leptin	32 weeks 36 weeks	+ r = 0.259 NS	+ + R^2^ = 0.146	The study population was pregnant adolescents. Leptin levels were positively correlated with GWG. The R^2^ value is indicative of the model that incorporates both leptin measurements.
Perichart-Perera et al. (2017) [7]	117 women	Leptin	11.42 ± 1.7 weeks	+ r = 0.235	+ β = 0.007	The multivariate association was significant only in normal-weight women.
		TNF-α	11.42 ± 1.7 weeks	− r = −0.196	− β = −14.99	The multivariate association was significant only in normal-weight women.
Hinkle et al. (2019) [8]	321 women	Leptin	33–39 weeks	ND	- β = −72.63	The significant associations observed in this study were adjusted for several maternal variables, including pBMI and GWG.
		Adiponectin	23–31 weeks 33–39 weeks	ND ND	− β = −38.14 − β = −25.96	See the previous comment for this same reference
Lekva et al. (2017) [9]	300 women	Adiponectin	Four measurements from 14–16 weeks (visit 1) to 36–38 weeks (visit 4)	ND	− β = −0.15	The multivariate association was determined with the adiponectin change from visit 1 to visit 4.
Lindberger et al. (2023) [10]	1349 women	Adiponectin	16–20 weeks	ND	− β= −17.1	The association was significant only in the unadjusted model.
Birth length (cm)						
Sámano et al. (2017) [6]	168 dyads	Leptin	32 weeks	+ r = 0.183	+ R^2^ = 0.085	See the previous comment for this same reference. The R^2^ value is from the model incorporating the leptin measurement.
Hinkle et al. (2019) [8]	321 women	sOB-R	33–39 weeks	ND	+ β = 0.07	See the previous comment for this same reference
Andersson-Hall et al. (2021) [11]	126 women	Leptin	Three measurements (8–12, 24–26, and 35–37 weeks)	ND	− β = −0.299 (T1–T2) β = −0.355 (T2–T3)	Neonatal length was measured at one week of age. The multivariate association was determined with the leptin change from trimester 1 to trimester 2, and from trimester 2 to trimester 3.
Birth head circumference (cm)						
Vernini et al. (2016) [5]	72 mother–newborn pairs	Adiponectin	37–38 weeks	− r = −0.27	NS	Adiponectin levels were negatively correlated with gBMI
Birth abdominal circumference (cm)						
Vernini et al. (2016) [5]	72 mother–newborn pairs	Leptin	37–38 weeks	− r = −0.25	SS	Leptin levels were positively correlated with pBMI.
Sum of skinfolds (mm) ♦						
Hinkle et al. (2019) [8]	321 women	sOB-R	10–14 weeks 33–39 weeks	ND ND	+ β = 0.11 + β = 0.11	See the previous comment for this same reference
		Free leptin index	15–26 weeks	ND	− β = −0.96	See the previous comment for this same reference
Fat mass (%)						
Radaelli et al. (2006) [12]	18 women	IL-6	Third trimester	+ r = 0.57	ND	No comments.
Andersson-Hall et al. (2021) [11]	126 women	Leptin	Three measurements (8–12, 24–26, and 35–37 weeks)	ND	− β = −0.318	Neonatal fat mass was measured at one week of age. The multivariate association was determined with the leptin change from trimester 2 to trimester 3 in normal weight women.

The plus signs indicate positive associations, and the minus signs negative ones. TNF-α: tumor necrosis factor-alpha; ND: not done; NS: not significant; SS: statistically significant (the original study does not indicate whether the association was positive or negative); pBMI: pregestational body mass index; gBMI: gestational (37–38 weeks of gestation) body mass index; GWG: gestational weight gain; sOB-R: soluble leptin receptor. ^♦^ Skinfold measurements were taken on the right side of the infant’s body at the abdominal flank, anterior thigh, subscapular, and triceps.
